# Dissolution of cellulose derivatives in NaOH/urea aqueous solvent and optimisation of a cationic cellulose/carboxymethyl cellulose hydrogel using response surface methodology

**DOI:** 10.1039/d5ra07141k

**Published:** 2026-01-21

**Authors:** Nur Fattima’ Al-Zahara’ Tuan Mohamood, Norhazlin Zainuddin, Sazlinda Kamaruzzaman, Hidayah Ariffin

**Affiliations:** a Department of Chemistry, Faculty of Science, Universiti Putra Malaysia 43400 Serdang Selangor Malaysia norhazlin@upm.edu.my; b Laboratory of Biopolymer and Derivatives, Institute of Tropical Forestry Product (INTROP), Universiti Putra Malaysia 43400 Serdang Selangor Malaysia

## Abstract

This study presents the synthesis and optimisation of a cellulose-based hydrogel derived from oil palm empty fruit bunch (OPEFB) waste *via* a sustainable green chemistry approach. Cationic cellulose (CC) was prepared through a cationisation using diallyl dimethyl ammonium chloride (DADMAC), followed by dissolution in a NaOH/urea aqueous system. A hydrogel was then synthesised by blending CC with carboxymethyl cellulose (CMC) and crosslinked with epichlorohydrin (ECH). Response surface methodology with a design model of central composite design (RSM/CCD) was applied to statistically optimise three key variables of CC : CMC ratio, ECH concentration and reaction temperature, for enhanced gel content and degree of swelling. The variables were modelled and analysed using quadratic Analysis of Variance (ANOVA). The optimum hydrogel formulation achieved a gel content of 20.95% and swelling capacity of 116.94 g g^−1^. FT-IR, XRD, TGA and FESEM-EDX were performed to characterize all samples in order to evaluate the success of the reaction optimisation. FT-IR spectroscopy confirmed successful chemical crosslinking of the hydrogel with ECH at 2913.07 cm^−1^ and 2879.77 cm^−1^, while XRD analysis revealed a transition from cellulose I to cellulose II with reduced crystallinity at 2*θ* = 20° and 2*θ* = 22° suggesting a higher degree of amorphous character. Thermal analysis of TGA demonstrated improved thermal stability in CC due to quaternary ammonium groups, whereas the hydrogel exhibited multi-stage degradation. FESEM-EDX micrographs revealed a highly porous morphology of the hydrogel with an average pore size of 81 µm. These findings validate the potential of cellulose for the development of biodegradable hydrogels with tunable physicochemical properties.

## Abstract 2

A sustainable cellulose hydrogel was developed through Response Surface Methodology optimisation by blending cationic cellulose with carboxymethyl cellulose, followed by epichlorohydrin crosslinking to achieve high gel content and swelling capacity.

## Graphical abstract

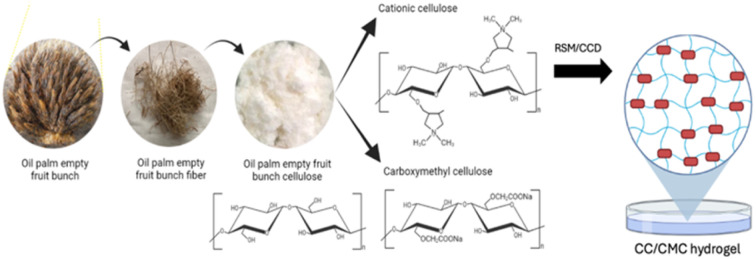

## 1. Introduction

As the world's second-largest exporter and producer of palm oil, behind Indonesia, Malaysia's development in oil palm plantations and palm oil milling has expanded rapidly year after year. The wide production and contribution of palm oil is attained by *Elaeis guineensis* due to its greater commercial value and high yield of high-quality oil compared to *Elaeis oleifera*.^1^ Despite its economic significance, the rapid expansion of the oil palm industry has led to significant biomass waste generation. Palm oil processing produces large amount of solid biomass waste, which, if not properly managed, can pose serious environmental concerns. Su *et al.* (2023) reported that more than 124 million tonnes of solid oil palm biomass waste were generated in 2021 alone from both palm oil mills and plantations.^2^ Hamzah *et al.* (2019) stated that the processed fresh fruit bunches by oil palm mills in 2017 were estimated at 101.02 million tonnes, with 51.19 million tonnes of total dry oil palm residues.^3^ The biomass wastes were approximately 27.13 million tonnes of fronds and trunks, 7.78 million tonnes of oil palm empty fruit bunches (OPEFBs), 8.18 million tonnes of mesocarp fibres, 4.72 million tonnes of palm kernel shells and 3.38 million tonnes of palm oil mill effluent.

The oil palm industry is linked to various environmental issues due to its land-intensive nature. Despite some efforts to process these wastes into organic fertilisers, a substantial amount of oil palm biomass waste remains unused, leading to challenges in waste disposal management. Traditional disposal approaches such as open burning are prohibited under Malaysia's zero-burning policy, reinforcing the need for sustainable waste management. Converting these residues into high-value materials represents a key strategy to support circular bioeconomy initiatives. The sustainability of the oil palm industry revolves around effective management, utilising oil palm residue to curtail the waste, increase profits and achieve sustainable progress. Hence, converting OPEFB into cellulose-based functional materials represents not only a waste valorisation strategy but also a necessity for achieving sustainable industrial practices.

Cellulose is by far the most plentiful organic polymer that exists on Earth and is regarded as an environmentally friendly material. Due to its biocompatibility and minimal environmental impact, cellulose is approved for various consumer products by the U.S. Food and Drug Administration (FDA) across various industries. Examples of FDA-approved cellulose derivatives include carboxymethyl cellulose, cellulose triacetate, diethylamino-cellulose, methyl ethyl cellulose and regenerated cellulose.^4^ This polysaccharide can be extracted from most agricultural crop waste, supporting zero-waste strategies. Naceur Abouloula *et al.* (2018) found that oil palm biomass waste, particularly OPEFB, contains a higher percentage of cellulose (44%) compared to other bioresources such as rice husk (35%), durian (33%) and bamboo cane (41%).^5^ This work employs high-cellulose OPEFB as a dual precursor for synthesising both cationic cellulose and carboxymethyl cellulose, a strategy that remains limited in the current literature. Hydrogels, particularly those derived from natural polymers, have gained significant interest due to their eco-friendly nature and ability to biodegrade. Hydrogels are generally defined as three-dimensional (3D) crosslinked polymer networks capable of absorbing large quantities of water due to the presence of hydrophilic functional groups.^6,7^ Hydrogel is also called as superabsorbent material as it absorbs and retains fluid up to 2000 g g^−1^ compared to its original dry weight in it network structure due to the available pores.^8^ The surrounding conditions that demonstrate changes in the hydrogel shape and volume either to swell or deswell, structural, permeability and mechanical adjustments are due to the various internal and external responses. Rizwan and his co-researchers (2017) reported that light, pressure, temperature, ultrasound and electric field are some examples of external stimuli, while pH, ionic strength, enzymes and antigens are the internal stimuli that may shift the original shape of the hydrogel.^9^

Derived from renewable polysaccharides, cellulose-based hydrogels present a sustainable green material. Cellulose has emerged as a highly suitable candidate for hydrogel fabrication, primarily owing to its inherent hydrophilic nature and biodegradable characteristics.^10^ They support a circular economy by efficiently utilising natural resources and reintegrating them into the environment. The development of cationic cellulose (CC) from OPEFB biomass waste and carboxymethyl cellulose (CMC) hydrogel further enhances their environmental benefits. With their non-toxic degradation profile, CC/CMC hydrogel holds great potential for applications in agriculture, water treatment and controlled-release systems. Their adoption can drive a transition toward greener technologies while promoting ecological responsibilities. The development of CC/CMC hydrogel from the OPEFB was conducted by using a statistical approach of response surface methodology (RSM). This approach is substituting the technique of the traditional optimisation one-variable-at-a-time (OVAT), which is more time-consuming. In contrast to prior works that often relied on conventional trial-and-error methods, this study employs RSM to systematically optimise reaction parameters, ensuring reproducibility, efficiency and performance enhancement of the hydrogel. This study develops and optimises a dual-charged CC/CMC hydrogel through a statistical design framework, *a* direction that has received limited attention while offering scientific and industrial relevance.

## 2. Experimental

### 2.1 Materials

The biomass waste of OPEFB was supplied by Jugra Palm Oil Mill Sdn. Bhd., Banting, Selangor Darul Ehsan, Malaysia. Carboxymethyl cellulose sodium salt (CMC, R&M Chemicals), diallyl dimethyl ammonium chloride (DADMAC, 65% wt., Sigma Aldrich), sodium hydroxide, (NaOH, 99%, Sigma Aldrich), ethanol (C_2_H_5_OH, 95%, Sigma Aldrich), hydrochloric acid (HCl, 32%, Sigma Aldrich), urea (CO(NH_2_)_2_, 99.5%) and epichlorohydrin (ECH, 99%) were of analytical grade and used without further purification. Distilled water was used throughout the experiment.

### 2.2 Preparation of CC from extracted cellulose of OPEFB

The preparation of CC from OPEFB was carried out according to Heinze *et al.* (2004) with some modifications.^11^ OPEFB cellulose was soaked in 1–25% (w/v) of 100 mL of NaOH solution and then heated and stirred for an hour at 60 °C in a water bath shaker. Then, 20–80% (w/v) DADMAC was added dropwise into the reaction mixture and heated for another 3–6 hours at 40–70 °C. The mixture was added with 200 mL of distilled water and was set aside to cool down before being neutralized with 1 M of HCl and 1 M of NaOH until neutral pH. Then, the cationic OPEFB cellulose was filtered and washed twice with 100 mL of ethanol and the final product was oven-dried at 40 °C until completely dried.

### 2.3 Dissolution of CC and optimisation of CC/CMC hydrogel

The dissolution of CC was conducted with CC added into 100 mL aqueous solvent of NaOH/urea/distilled water at the weight percent ratio of 7 : 12 : 81 (w/w) and the mixture was pre-cooled at a low temperature of −10 °C for 24 hours. The mixture was stirred for 10 minutes and was observed under an optical microscope. CC/CMC hydrogel was optimised using a statistical approach of RSM based on a CCD model with three variables: the ratio of CC (4 : 6, 5 : 5, and 6 : 4 (w/w)), the concentration of ECH (5, 7.5, and 10 w/v), and the reaction temperature (40 °C, 50 °C, 60 °C). Hydrogel was produced by blending CMC with optimised CC. CMC powder was introduced into the CC solution and stirred until complete dissolution was achieved. Epichlorohydrin (ECH), serving as the crosslinking agent, was added to the CC/CMC mixture and stirred at room temperature. The resulting CC/CMC hydrogel paste was then placed in an oven at 40–60 °C for 15 hours to facilitate crosslinking. A small portion of the hydrogel was subsequently sampled for the determination of gel content and swelling ratio.

### 2.4 Gel content and degree of swelling of CC/CMC hydrogel

Gel content and degree of swelling of the hydrogel were studied by referring to Fei *et al.* (2000) and Zhang *et al.* (2020).^12,13^ The gel content of the CC/CMC hydrogel was determined by measuring the insoluble part after the immersion of the hydrogel in distilled water for 72 hours at room temperature. The distilled water was replaced every 24 hours. The percentage of gel content was calculated using the following equation:

1 Percentage of gel content = *W*_da_/*W*_db_ x 100%

where *W*_da_ is the weight of dried hydrogel after immersion and *W*_db_ is the weight of hydrogel before immersion.

The degree of swelling of CC/CMC hydrogel was determined by the immersion of the hydrogel in distilled water for 72 hours at room temperature. The hydrogel was weighed after it reached equilibrium, and the degree of swelling was calculated using the following equation:

2 Degree of swelling = (*W*_s_ − *W*_d_)/*W*_d_

where *W*_s_ is the weight of swollen hydrogel and *W*_d_ is the weight of the dried hydrogel.

### 2.5 Central composite design prediction and optimisation of process variables

The preparation of CC/CMC hydrogel was optimised using a mathematical approach with the aid of experimental design methodology, which is RSM. RSM is one of the popular designs of experiment (DoE) approach used to study all variables at once to obtain optimal results. The experiment was conducted based on the Central Composite Design (CCD) and constructed using the Design-Expert Software version 13.0.1 Stat Ease Inc., Minneapolis, Minnesota, USA. The design model of RSM/CCD can correctly describe the experimental data based on adequate correlation.^14^ Three variables were studied: 1) ratio of CC : CMC (w/w); 2) concentration of ECH; and 3) reaction temperature.^15^

A face-centred and orthogonal quadratic central composite design, RSM/CCD framework was designed based on the independent variable with 20 sets of experiments. The approach of Analysis of Variance (ANOVA) was used to predict the optimum value of gel content and degree of swelling. The design of RSM/CCD, which comprises linear, quadratic and interaction factors, was proposed with a quadratic expression as shown in eqn (3)^15^3
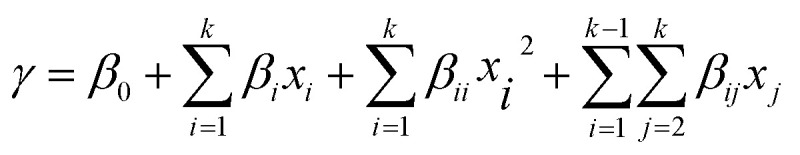
where *γ* refers to the predicted responses, *β*_0_ is the constant coefficient, *β*_*i*_ is the *i*th linear coefficient, *β*_*ii*_ is the quadratic coefficient, *β*_*ij*_ is the *ij*th interaction coefficient while *x*_*i*_ and *x*_*ij*_ represent the factors. The response surface analysis of the combined variables indicates the variable's optimal value. By introducing the desired variables to the software, the constraints in this study were used to estimate the best variable conditions that would yield the highest gel content and degree of swelling. By carrying out the suggested experiments and contrasting the outcomes with the predictions, the accuracy RSM/CCD design model was confirmed. The reliability of the model was assessed using residual standard error (RSE). Based on the second-order polynomial model, the numerical optimization of the response was estimated.

The number of trials for RSM/CCD design model is composed of N as the total number of trials or experiments and standard 2^*n*^ factorial points with the origin at the centre. The axial point of 2*n* is constant at all points equidistant from the design centre while *n*_c_, denotes the number of central points that have replicates of the trials at the centre and is very crucial in offering an independent estimation of the experimental error.^16,17^ The number of this optimized RSM/CCD design model trials was calculated based on eqn (4).

4 *N* = 2^*n*^ + 2*n* + *n*_c_ = 2^4^ + (2 x 4) + 6 = 30

### 2.6 Characterisation of CC/CMC hydrogel

FTIR spectra of the samples were recorded using an attenuated total reflectance (ATR) fourier transform infrared nicolet is 10 (Thermo Fisher Scientific, USA) in the range of 500–4000 cm^−1^ at an average of 16 scans with a resolution of 4 cm^−1^. XRD characterization was carried out using PW 3040/60 MPD X'pert High Pro PANalytical diffractometer (Philips, Netherlands) with Cu-Kα (*λ* = 1.5418 Å) radiation at room temperature and operated at 40 kV and 40 mA with a step size of 0.08. A sample was mounted on a sample holder, and the diffraction pattern was recorded by plotting intensity as a function of the detector angle (2*θ*) over a scanning range of 5°–50° in continuous scan mode. The Miller indices were provided by the Mercury program for all peaks. The indices of the peaks were indicated in blue for cellulose Iβ and red for cellulose II. The XRD diffractogram was plotted with the aid of OriginPro 2019 Graphing and Analysis software. TGA was performed using a minimum of 1 mg samples which were heated from 50–800 °C at a rate of 10°C min^−1^ under an ultra-pure nitrogen atmosphere using Mettler Toledo TGA/SDTA-851^e^/STAR^e^. The TGA thermal curve is displayed as temperature (*X*-axis) and weight percent (%) (*Y*-axis). Surface morphology and elemental analysis of untreated OPEFB and its derivatives were examined with FESEM-EDX (JEOL, JSM-7600F). = The micrographs of all samples were captured at an accelerating voltage of 5 kV, using various magnifications. Each sample was separately sprinkled onto the sample holder and coated with platinum before analysis. All hydrogel samples were freeze-dried prior to TGA, XRD, and FESEM analyses to ensure structural stability and avoid interference from free water.

## 3. Results and discussion

### 3.1 Dissolution of CC

**Fig. 1:**
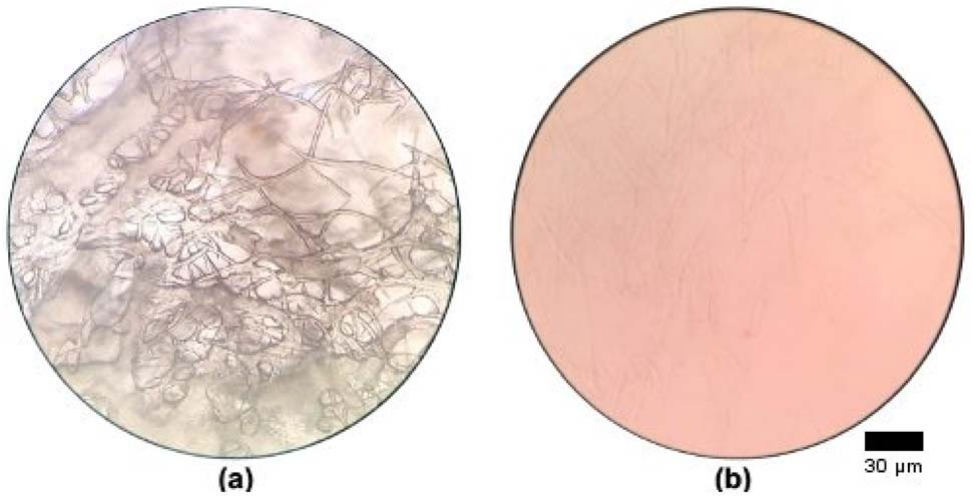
Image of (a) swollen and undissolved CC and (b) dissolved CC under optical microscopy.

Cellulose, a common natural polymer, is notoriously difficult to process due to its crystalline structure and extensive network of strong inter- and intramolecular hydrogen bonds. The intricate hydrogen bond network contributes to its limited solubility in liquid solvents, resulting in high resistance to dissolution. In this study, CC was successfully dissolved in aqueous NaOH/urea solution and subsequently observed under an optical microscope, as presented in Fig. 1.

Fig. 1 highlights the influence of temperature on the dissolution behaviour of CC, where limited dissolution is observed at room temperature (27 °C, Fig. 1(a)), while markedly improved dissolution is evident at the lower temperature of −10 °C (Fig. 1(b)).The dissolution of CC at lower temperature is likely due to the formation of an inclusion complex between cellulose, NaOH and urea hydrates. Xiong *et al.* (2014) found that adding urea to a NaOH solvent helps mitigate the hydrophobic effect of cellulose.^18^ In this solvent system, hydrated Na^+^ from NaOH disrupts hydrogen bonds between the cellulose chains and interacts with the hydrophilic hydroxyl groups. Concurrently, urea interacts with the hydrophobic regions of cellulose. This combined action enhances the cellulose solubility in the aqueous solvent and stabilises the solution by preventing hydrophobic parts from reassociating.

Wang & Deng (2009) highlighted that temperature plays a crucial role in cellulose dissolution within the NaOH/urea aqueous solvent system.^19^ Since cellulose dissolution is an exothermic reaction, it is more efficient at a lower temperature.^20^ At these cooler conditions, a robust network of solvent hydrates helps break down cellulose chains by forming new hydrogen bonds with cellulose and NaOH hydrates. Simultaneously, urea attaches to the hydroxyl groups of cellulose *via* both inter- and intramolecular hydrogen bonds, effectively stopping the cellulose chains from clumping together.

### 3.2 Reaction mechanism of CC/CMC hydrogel

**Fig. 2:**
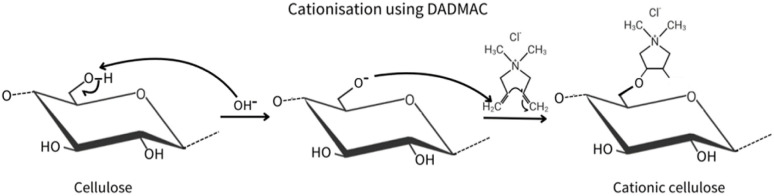
Reaction mechanisms of cellulose modification using DADMAC.

OPEFB cellulose was first modified into CC, as illustrated in Fig. 2, using DADMAC in the presence of NaOH as a catalyst. The NaOH deprotonated the C6 hydroxyl groups of cellulose, generating reactive alkoxide ions. DADMAC, acting as an electrophile, was then attacked by these cellulose alkoxide ions (nucleophiles) at its double bonds, forming a covalent bond. This substitution can occur at C2, C3 or C6 positions.^21^ Finally, DADMAC molecules lost a chloride ion, forming a quaternary ammonium cation that introduced positive charges onto the cellulose backbone.

**Fig. 3:**
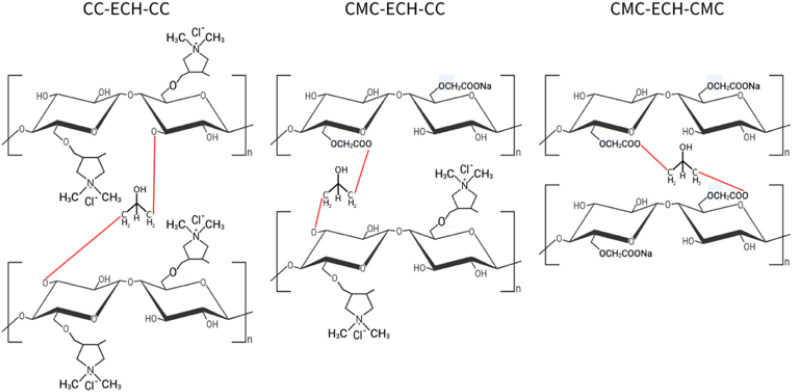
Proposed chemical structures of CC/CMC hydrogel.

Both CC and CMC were dissolved in an aqueous solvent of NaOH/urea, then crosslinked with ECH. ECH containing epoxide and chloroalkyl groups, enables a combination of physical and chemical interactions with CC/CMC, leading to simultaneous ether bond formation and chain entanglement.^22^Fig. 3 illustrates three potential crosslinked polymer structures formed between ECH and CC/CMC. The reaction between ECH and CC formed a chlorohydrin anion. The lone pair electron of the hydroxyl groups of the CC at C3 position, which serves as a nucleophile, attacked the chlorohydrin anion of the carbon atom. This cleavage of the C–Cl bond resulted in the formation of ether linkages between the cellulose chains and ECH molecules.

For the ECH-CMC reaction, NaOH deprotonates the hydroxyl group of CMC into nucleophilic alkoxide ions. These ions then attack the carbon atom linked to the chlorine in ECH, likely at C6, breaking the C–Cl bond and forming an ether linkage between ECH and CMC. Salleh *et al.* (2019) observed that OPEFB cellulose crosslinked with ECH primarily at C3 and C2 reacting with Na^+^, while OPEFB CMC favoured crosslinking at C6 due to less steric hindrance.^23^ This covalent C–O–C bond formation during crosslinking helps the hydrogel regenerate and allows the solution's gelation to form a stable network.

#### 3.2.1 Statistical analyses and model fitting of model design equation

The synthesis of CC/CMC hydrogel was accomplished using the statistical design methodology of RSM using CCD with a quadratic design model. Table 1 shows the full design model with predicted and actual gel content and degree of swelling of the CC/CMC hydrogel. The response of RSM/CCD is the actual gel content and degree of swelling, ranging from 16.47–23.92% and 22.76–140.12 g g^−1^, respectively, across the 20 experimental runs. As reported by Omorogie *et al.* (2017), the experimental error was resolved with six repetitions at the centroid, which were experiment numbers 1, 7, 10, 11, 12 and 13.^24^ The ratio of maximum to minimum actual value of gel content, according to the design model, is 1.45, whereas the degree of swelling is 6.16, both indicate a good ratio value and a ratio greater than 10 usually requires some model transformation. However, a ratio value less than 3 indicates that the power transformations have little effect. Hence, no adjustment is needed. The following equation defines the empirical formula for both gel content and degree of swelling of the CC/CMC hydrogel in terms of coded factor:Gel content = 20.74–0.3330*A* + 2.24*B*–0.5810*C*Degree of swelling = 42.68–11.76*A*–33.71*B* + 10.82*C* + 0.9314*A*^2^ + 13.43*B*^2^ + 7.40*C*^2^ + 6.35A*B*–4.80*AC*–7.07*BC*where, *A* = ratio of CC : CMC, *B*=concentration of ECH, *C*=reaction temperature.

**Table 1:** Design summary of experimental value of gel content and degree of swelling for CC/CMC hydrogel

sample run	Variables			Gel content		Reaction efficiency (%)	Degree of swelling		Reaction efficiency (%)
	*A*: ratio of CC : CMC	*B*: ECH	*C*: reaction temperature	Predicted	Experimental		Predicted	Experimental	
1	5 5	7.5	50	20.28	20.80	100	42.68	44.97	100
2	4 6	5	60	19.00	19.46	100	138.95	140.12	100
3	6 4	10	40	23.98	23.30	97.16	26.37	25.29	95.90
4	6 4	5	40	19.45	20.34	100	66.95	66.18	98.85
5	5 5	7.5	60	20.55	21.41	100	60.90	56.35	92.53
6	4 6	7.5	50	21.08	21.94	100	55.37	55.67	100
7	5 5	7.5	50	20.28	20.69	100	42.68	54.77	100
8	4 6	5	40	20.25	19.57	96.64	93.56	95.17	100
9	6 4	7.5	50	20.41	20.41	100	31.86	31.19	97.90
10	5 5	7.5	50	20.28	19.43	95.81	42.68	42.24	98.97
11	5 5	7.5	50	20.28	19.84	97.83	42.68	47.41	100
12	5 5	7.5	50	20.28	19.49	96.10	42.68	36.47	85.45
13	5 5	7.5	50	20.28	19.68	97.04	42.68	30.98	72.59
14	4 6	10	60	23.44	22.33	95.26	44.69	45.55	100
15	6 4	5	60	17.15	16.47	96.03	93.15	97.18	100
16	5 5	5	50	17.64	17.64	100	89.82	83.78	93.38
17	6 4	10	60	22.91	23.37	100	24.27	22.76	93.78
18	5 5	7.5	40	21.71	21.72	100	39.26	43.44	100
19	4 6	10	40	23.46	23.92	100	27.60	23.66	85.72
20	5 5	10	50	22.12	22.98	100	22.41	28.08	100

As shown in Table 2, for gel content, the suggested linear model proved more significant (*p* < 0.0001) than the proposed cubic model (*p* = 0.1931). A low *p*-value indicates the model's optimisation by linear terms. Thus the linear model was chosen for the gel content of the CC/CMC hydrogel. Conversely, for the degree of swelling in Table 3, the quadratic model was more significant (*p* = 0.0010) compared to the cubic model (*p* = 0.6568). With its *p*-value also below 0.0500, the quadratic model was selected for the degree of swelling of the hydrogel.

**Table 2:** Fit summary for gel content of CC/CMC hydrogel

source	*p*-value sequential	*p*-value lack of fit	Adjusted *R*^2^	Predicted *R*^2^	
Linear	<0.0001	0.0929	0.7371	0.6143	Suggested
2FI	0.5657	0.0734	0.7218	−0.0779	
Quadratic	0.1997	0.0888	0.7680	−0.5079	
Cubic	0.1931	0.0831	0.8407	−28.8767	Aliased

**Table 3:** Fit summary for degree of swelling of CC/CMC hydrogel

Source	*p*-value sequential	*p*-value lack of fit	Adjusted *R*^2^	Predicted *R*^2^	
Linear	<0.0001	0.0747	0.7726	0.6431	
2FI	0.2277	0.0850	0.7971	0.1399	
Quadratic	0.0010	0.8160	0.9451	0.8947	Suggested
Cubic	0.6568	0.9492	0.9357	0.9515	Aliased

#### 3.2.2 Analysis of Variance (ANOVA) of optimisation of CC/CMC hydrogel

The ANOVA results for CC/CMC hydrogel optimization, as presented in Table 4, indicated significant F-values for both gel content (18.76%) and degree of swelling (37.43 g g^−1^), indicating a less than 0.01% chance these values occurred randomly. The *p*-values for both responses were less than 0.0500, thereby confirming the significance of the model. Both values indicate that the terms show a good guidelines and have significant effects on the responses.^25^

**Table 4:** ANOVA for response surface model for CC/CMC hydrogel

Source	Gel content						Degree of swelling					
	SOS	DOF	Mean square	*F*-value	*P*-value		SOS	DOF	Mean square	*F*-value	*P*-value	
Model	54.75	3	18.25	18.76	<0.0001	Significant	16 715.25	9	1857.25	37.34	<0.0001	Significant
A-ratio of CC : CMC	1.11	1	1.11	1.14	0.3015		1382.27	1	1382.27	27.79	0.0004	
B-ECH	50.27	1	50.27	51.67	<0.0001		11 362.97	1	11 362.97	228.43	<0.0001	
C-temperature	3.38	1	3.38	3.47	0.0810		1171.16	1	1171.16	23.54	0.0007	
A^2^	—	—	—	—	—		2.39	1	2.39	0.0480	0.8311	
B^2^	—	—	—	—	—		496.10	1	496.10	9.97	0.0102	
C^2^	—	—	—	—	—		150.44	1	150.44	3.02	0.1127	
AB	—	—	—	—	—		322.20	1	322.20	6.48	0.0291	
AC	—	—	—	—	—		184.03	1	184.03	3.70	0.0833	
BC	—	—	—	—	—		400.30	1	400.30	8.05	0.0176	
Lack of fit	13.74	11	1.25	3.42	0.0929	Not significant	148.10	5	29.62	0.4239	0.8160	Not significant
Pure error	1.83	5	0.3657				349.34	5	69.87			
*R* ^2^	0.7786						0.9711					
Adjusted *R*^2^	0.7371						0.9451					
Predicted *R*^2^	0.6143						0.8947					
Adequate precision	14.3099						23.3683					
Standard deviation	0.9863						7.05					
CV (%)	4.76						13.17					

#### 3.2.3 Validation and verification of design model

For gel content, the factor *B* (crosslinking agent concentration) was the most significant, outweighing the factor *A* (CC : CMC ratio) and factor *C* (reaction temperature). For the degree of swelling, factors *A*, *B*, *C*, *AB*, *BC* and *B*^2^ were all significant. This suggests swelling is not controlled by a single factor but by a complex interplay of these variables. While other coefficient terms were not highly significant, they were kept for the model hierarchy.

**Fig. 4:**
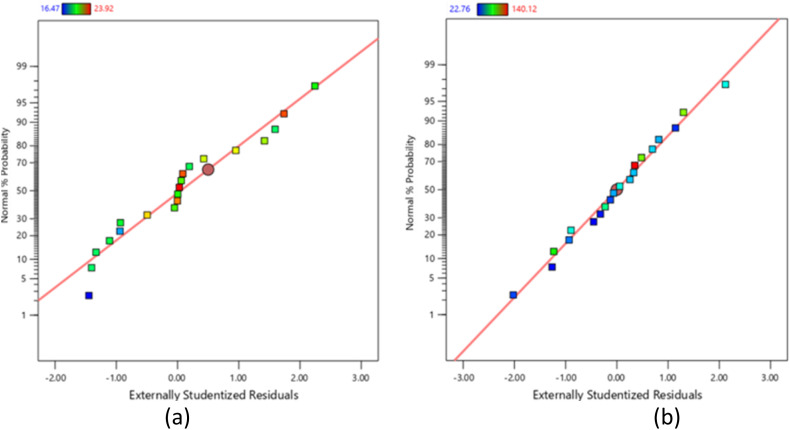
Graph of normal plot of residuals for (a) gel content and (b) degree of swelling of CC/CMC-com hydrogel.

The normal probability plots of residuals in Fig. 4 for both gel content and degree of swelling closely followed a straight line, confirming the accuracy of the RSM/CCD model in predicting the surface response. The degree of swelling plot showed a better distribution, indicating more fitted values, whereas the gel content plot had a slight “S-shaped” curve with slightly more residual values. Crucially, no residuals significantly deviated from the normal probability line. This supports the findings of Mustafa *et al.* (2019), who likewise reported that their RSM/CCD model effectively predicted the surface response when the residuals showed no significant deviation from the response line.^26^ Normal distribution in their study on optimising PVA/TiO_2_ nanofiber efficiency.

**Fig. 5:**
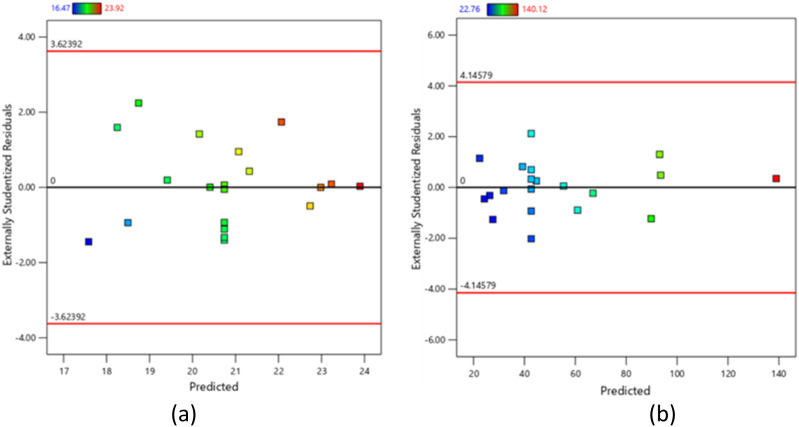
Graph of residuals *versus* predicted for (a) gel content and (b) degree of swelling of CC/CMC hydrogel.

Fig. 5 displays randomly spread residual data points for predicted gel content and degree of swelling, indicating even distribution around the mean of RSM/CCD model. For gel content, the maximum and minimum limits are between +3.62392 and −3.62392, respectively. Meanwhile, for degree of swelling, the limits of the model are between +4.14579 and −4.14579. Since all data points for both responses fall within these ranges, the RSM/CCD model is accurate, lacks constant error and contains no potential outliers.

**Fig. 6:**
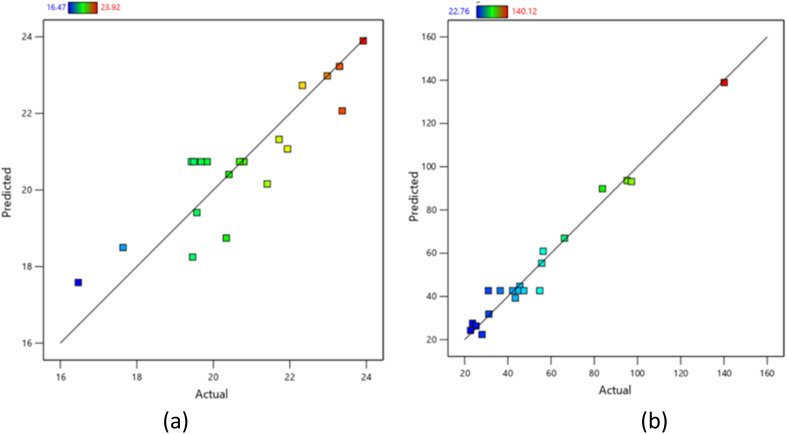
Correlation between predicted *versus* actual for (a) gel content and (b) degree of swelling of CC/CMC hydrogel.

Fig. 6 illustrates the relationship between predicted and actual values for both gel content (a) and degree of swelling (b) of the CC/CMC hydrogel. The main goal of these plots is to identify any values that the RSM/CCD model might not predict well. In both graphs, the data points are quite close to the straight line and each other, indicating a linear relationship between the predicted and actual values. This arrangement suggests a highly accurate data plot. The closer the actual values are to the predicted values of the RSM/CCD model, the more reliable and predictable the model becomes.

#### 3.2.4 Response surface and contour plot

**Fig. 7:**
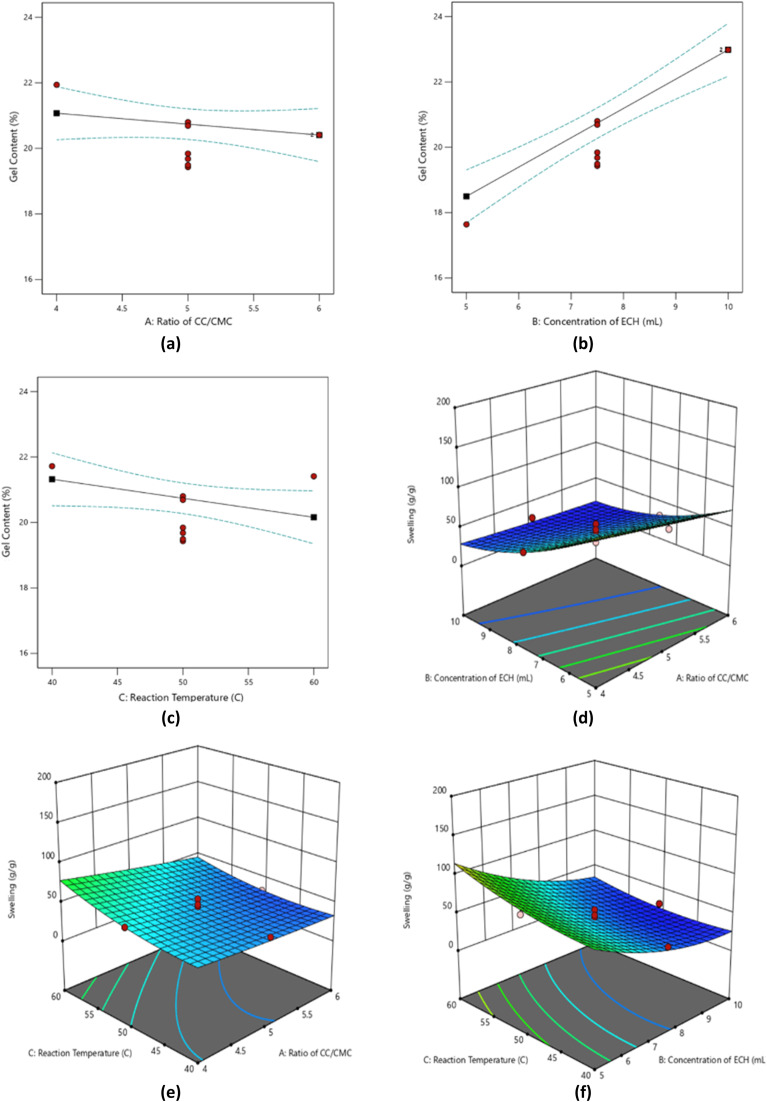
(a–c) Single factor that affects the gel content and (d–f) simultaneous factors that affect the degree of swelling of CC/CMC-com hydrogel.

2D and 3D contour plots in Fig. 7 illustrate the correlation between independent variables on both gel content and degree of swelling of CC/CMC hydrogel.

##### 3.2.4.1 Effect of CC : CMC ratio on gel content

Fig. 7a presents the effect of varying CC : CMC ratios on the gel content of the CC/CMC hydrogel. Based on the 2D response plot, the variations in the CC : CMC ratio do not significantly alter the gel content. This limited effect is likely attributable to the restricted solubility of both CC and CMC in the NaOH/urea solvent system, which contains the range of achievable ratios. Exceeding the solubility threshold may lead to incomplete dissolution, thereby compromising the homogeneity and overall efficiency of the crosslinking reaction.

According to the RSM/CCD model, broader ranges in the CC : CMC ratio would potentially yield more pronounced differences in hydrogel performance. However, due to solubility constraints, the experimental ratios were confined to 4 : 6, 5 : 5 and 6 : 4. Gel content values of 21.07%, 20.80%, and 20.41% were recorded, respectively.These ratios were selected in accordance with the maximum dissolution capacity of the modified cellulose materials, which is limited to 10% (w/v) due to the narrow range of NaOH concentrations effective for cellulose solubilisation in the NaOH/urea aqueous system.

This observation aligns with previous findings by Egal *et al.* (2008), who reported that the solubility limit of microcrystalline cellulose in NaOH/water mixtures does not exceed 10%.^27^ Within these limitations, the optimal CC : CMC ratio determined by the RSM/CCD model was 4 : 6, which resulted in the highest gel content of 21.07%.

##### 3.2.4.2 Effect of ECH concentration on gel content

Fig. 7b illustrates the effect of the crosslinking agent, ECH concentration, on the gel content of CC/CMC hydrogel. Increasing the ECH concentration from 5% (w/v) to 10% (w/v) resulted in a corresponding rise in gel content from 18.50% to 22.98% This can be attributed to the increased availability of reactive sites on the cellulose derivatives, allowing for more extensive chemical crosslinking. As the ECH concentration increases, the formation of hydrogen bonds between cellulose derivative chains is promoted, thereby reinforcing the polymeric network and enhancing hydrogel stability.

This finding is consistent with Bendoraitiene *et al.* (2018), who demonstrated that the degree of crosslinking in ECH-crosslinked cationic starch systems is strongly dependent on the concentration of the crosslinking agent; higher concentrations result in increased crosslink density.^28^ The present study limits the ECH concentration range to 5–10% (w/v), in line with previous research employing 5% and 7% ECH concentrations for fabricating cellulose-based hydrogels for various industrial applications.^29,30^

Furthermore, as reported by Muharam *et al.* (2017) in their study of starch-based hydrogels, higher ECH concentrations were found to reduce the water absorption capacity, suggesting a trade-off between crosslink density and swelling behaviour.^31^ Taking these factors into consideration, the optimum ECH concentration determined in this study was 10% (w/v), which yielded the highest gel content of 22.98%.

##### 3.2.4.3 Effect of reaction temperature on gel content

Fig. 7c indicates the influence of reaction temperature as an independent variable on the gel content. The reaction temperature was varied between 40 °C and 60 °C during hydrogel synthesis. A slight decrease in gel content was observed, from 21.32% at 40 °C to 20.16% at 60 °C. This is likely attributed to the partial disentanglement and degradation of the cellulose polymer network during the crosslinking process at elevated temperatures. At higher temperatures, the structural integrity of the cellulose may be compromised, leading to reduced crosslinking efficiency.

Similar findings were reported by Butrim *et al.* (2020), who observed that elevated temperatures during the cationisation of cellulose in an alkaline environment disrupted the polymer structure.^32^ In the present study, the preparation of the CC/CMC hydrogel involved the use of NaOH during the dissolution stage. Yue *et al.* (2015) further supported this phenomenon, noting that NaOH disrupts the inter-fibrillar regions of cellulose, and when combined with higher temperatures, may significantly promote fibril disentanglement.^33^ These findings suggest that while moderate heat may facilitate reaction kinetics, excessive thermal exposure during the presence of alkaline conditions can hinder effective crosslinking by weakening the cellulose framework.

##### 3.2.4.4 Effect of CC : CMC ratio and ECH concentration on degree of swelling

The simultaneous effect of the CC : CMC ratio and ECH concentration on the degree of swelling of CC/CMC hydrogel is shown in the 3D contour plot in Fig. 7d. It shows that the swelling capacity of the hydrogel increases with a decrease in ECH concentration and variation in CC : CMC ratio. This increment is attributed to the ability of the hydrogel to absorb more water due to the formation of a flexible network with larger pore sizes. The main factor of this behaviour is the lower concentration of the ECH, which results in reduced crosslinking density. Consequently, fewer hydrogen bonds form along the cellulose backbone, allowing more water to penetrate and occupy the available pore spaces within the hydrogel matrix. This aligns with findings by Wei & Chu (2014), who reported that the swelling of poly(*N*,*N*-dimethylaminoethylmethacrylate) (PDMAEMA) microcapsules is strongly influenced by the crosslinking agent concentration.^34^ Lower crosslinking density yields a more elastic network with expanded pores, enhancing the microcapsules' water absorption and resulting in a higher swelling ratio.

Among the tested formulations, the CC : CMC with 4 : 6 ratio yielded the highest swelling, outperforming other ratios. The enhanced swelling is attributed to the higher CMC content. The increased presence of anionic functional groups of carboxyl contributes significantly to water uptake. Supporting this, preliminary observations revealed that hydrogels made solely from CMC exhibited greater swelling capacity than those based on CC, despite both being crosslinked using the same agent. This is consistent with the findings of Spaic *et al.* (2014), who reported that anionic bacterial cellulose (BC) hydrogels retained water up to more than 15 000% at pH 7, compared to less than 4000% for cationic BC.^35^ This difference is largely due to the water-attracting nature of the carboxyl (-COOH) groups in anionic BC, which enhance water absorption and retention.

In conclusion, the optimal swelling performance of the CC/CMC-com hydrogel was achieved at an ECH concentration of 5% (w/v) and a CC : CMC ratio of 4 : 6, yielding a maximum swelling capacity of approximately 108.68 g g^−1^.

##### 3.2.4.5 Effect of CC : CMC ratio and reaction temperature on degree of swelling

Fig. 7e presents the combined influence of the CC : CMC ratio and reaction temperature on the degree of swelling. These two variables significantly affect the swelling behaviour of the CC/CMC hydrogel. As the temperature is increased from 40 °C to 60 °C and the CC : CMC ratio varied from 6 : 4 to 4 : 6, the swelling increased markedly from approximately 33 g g^−1^ to 75 g g^−1^. This is likely due to the partial disentanglement of the cellulose network at elevated temperatures during the crosslinking process. This has disrupted the polymeric structure, reducing crosslinking efficiency and increasing the mobility of polymer chains, thereby allowing more water molecules to penetrate and bind within the network.

This phenomenon is supported by Jayaramudu *et al.* (2019), who observed a similar trend in cellulose nanocrystal (CNC)-based polyacrylamide hydrogels synthesized *via* radical polymerisation.^36^ Their findings demonstrated that higher temperatures weaken hydrogen bonding among polymer chains, loosening the network structure. The increase in water uptake due to the action of capillary forces, osmotic pressure and other molecular interactions, ultimately resulting in greater swelling.

In contrast, when CC : CMC ratio shifts from 4 : 6 to 6 : 4, swelling decreases from 47 g g^−1^ to 33 g g^−1^. This reduction is attributed to the decreased proportion of CMC in the hydrogel formulation. CMC contains negatively charged carboxymethyl (–COO^−^) groups, which are highly hydrophilic and form strong hydrogen bonds with water molecules. A lower CMC content leads to reduced hydrophilicity of the hydrogel, diminishing its capacity to absorb and retain water.

This observation is further supported by Uyanga & Daoud (2021), who investigated the swelling behavior of carboxymethyl cellulose–chitosan composite hydrogels.^37^ They concluded that the high concentration of COOH and OH groups in CMC enhances water uptake through strong electrostatic interactions, effectively doubling the swelling ratio. In contrast, increasing chitosan content limits water absorption due to the formation of ionic crosslinks, which restrict polymer chain mobility and hinder swelling.

##### 3.2.4.6 Effect of ECH concentration and reaction temperature on degree of swelling

Fig. 7f depicts the combined effects of ECH concentration and reaction temperature on the degree of swelling of the hydrogel. The 3D contour plot indicates that reaction temperature has a more pronounced influence on swelling behavior than ECH concentration. As the reaction temperature increases from 40 °C to 60 °C, the degree of swelling rises from 79 g g^−1^ to 110 g g^−1^. This enhancement is attributed to the greater thermal energy at elevated temperatures, which increases the mobility of cellulose polymer chains. The thermal motion weakens intermolecular hydrogen bonds, resulting in a more flexible and expanded network structure that facilitates greater water uptake.

This observation is supported by Shan *et al.* (2023), who reported similar behaviour in nanocrystalline cellulose (NCC) ionogels.^38^ They found that elevated temperatures disrupted hydrogen bonding within the gel matrix, which increased the mobility of both polymer chains and NCCs, thereby enhancing swelling. In contrast, increasing the ECH concentration from 5% to 10% leads to a decrease in swelling capacity from 79 g g^−1^ to 26 g g^−1^. This inverse relationship is attributed to a higher degree of crosslinking. With greater availability of CC and CMC functional groups, higher ECH concentrations promote the formation of more crosslinking points, resulting in a denser and rigid polymeric network. The denser structure restricts water penetration by reducing pore size and free volume within the hydrogel.

These results are consistent with the findings of Fekete *et al.* (2014), who studied superabsorbent hydrogels crosslinked with *N*,*N*′-methylene-bis-acrylamide (MBA).^39^ Their work demonstrated that increasing the concentration of the crosslinking agent led to a higher gel fraction but reduced swelling capacity due to the formation of tighter, less porous network structures. Similarly, in the present study, higher ECH concentrations increased the crosslinking density, thereby reducing the hydrogel's swelling capacity by restricting water diffusion into the compact network.

#### 3.2.5 Validation of gel content and degree of swelling of CC/CMC hydrogel by RSM modelling

Three confirmation experiments were conducted, as detailed in Table 5, to evaluate the accuracy of the predicted values and validate the robustness of the established RSM/CCD design model. The primary objective was to obtain the maximum gel content and degree of swelling for the CC/CMC-com hydrogel formulation. Although these three confirmation runs were not part of the initial 20 experimental runs used in model development, they were performed within the same previously defined parameter ranges.

**Table 5:** Predicted and experimental response gel content and degree of swelling conducted at an optimum combination for CC/CMC hydrogel^a^

No.	CC : CMC ratio (%)	ECH (%)	Reaction temp. (°C)	Gel content		RE (%)	RSE (%)	Degree of swelling		RE (%)	RSE (%)
				Predicted	Actual			Predicted	Actual		
1	4 6	6.058	60	20.202	20.95	100	3.70	110.048	116.94	100	6.26
2	4 6	5.601	60	19.699	16.92	85.89	14.11	121.942	167.34	100	37.23
3	4 6	6.286	60	20.443	20.10	98.32	1.68	104.447	89.93	86.93	13.90

a RE: Reaction Efficiency; RSE: Residue Standard Error.

The results of the confirmation runs, along with the model validation and optimisation data presented in the table, demonstrated strong agreement between predicted and actual outcomes. All three experiments exhibited relative error (RE) exceeding 85% and relative standard errors (RSE) below 15%, indicating high predictive accuracy of the RSM/CCD model. Notably, experiment 1 showed perfect correlation, with 100% RE for both gel content and degree of swelling, achieving values of 20.95% and 116.95 g g^−1^, respectively. Therefore, among the confirmation trials, experiment 1 is identified as the optimal formulation condition for synthesising CC/CMC hydrogel, as predicted by the RSM/CCD design model.

### 3.3 FT-IR analysis of CC/CMC hydrogel

**Fig. 8:**
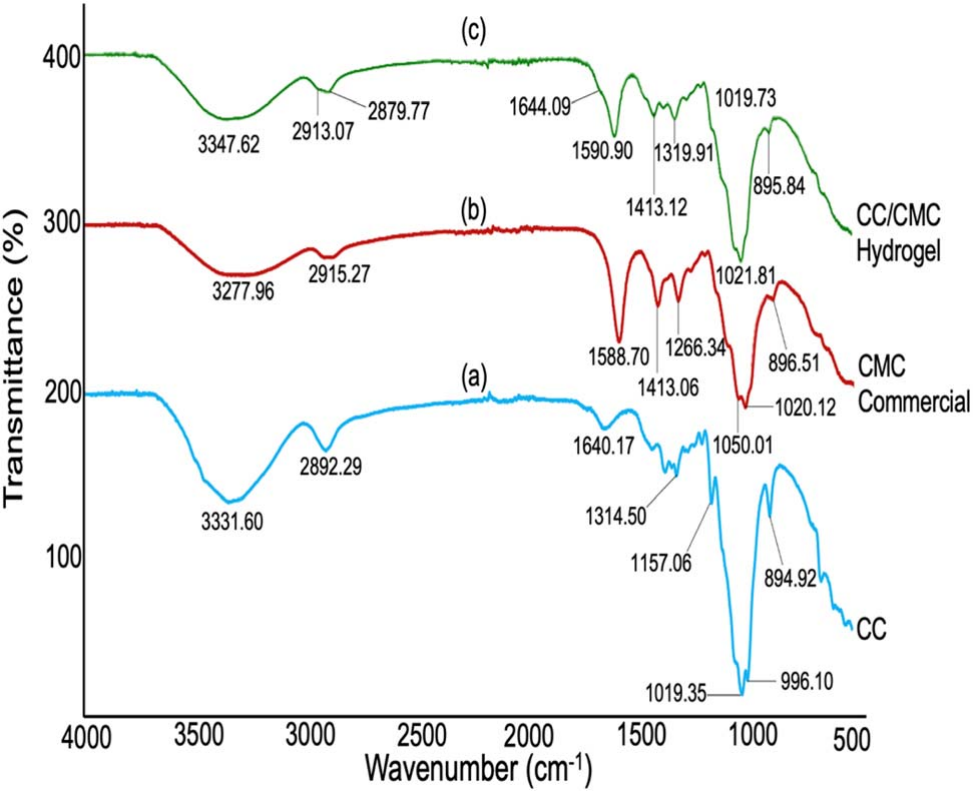
FT-IR spectra of (a) CC, (b) CMC and (c) CC/CMC hydrogel with CC : CMC ratio of 4 : 6.

FT-IR analysis was conducted to investigate the chemical modifications of native cellulose and to identify the formation of new functional groups following cationisation, carboxymethylation, and crosslinking reactions. The FT-IR spectra in Fig. 8 present the profiles of the cellulose derivatives—cationic cellulose (CC), carboxymethyl cellulose (CMC), and the crosslinked CC/CMC hydrogel. The spectral region between 1800–4000 cm^−1^ corresponds to the group frequency region, while the fingerprint region lies between 500–1800 cm^−1^. The polysaccharide structure, composed of glucose units, is indicated by the broad absorbance band in the range of 3200–3700 cm^−1^, corresponding to O–H stretching vibrations. Additionally, the shoulder peak observed between 2750–3000 cm^−1^ represents C–H stretching vibrations associated with intra- and intermolecular hydrogen bonding within the glycosidic linkages of the cellulose backbone.

Fig. 8a shows the spectrum of CC after cationisation using the quaternary ammonium reagent, DADMAC. A distinct new peak at 996.10 cm^−1^ is assigned to the CH_2_ group attached to the quaternary ammonium functional group of the cationic reagent, DADMAC. This result is consistent with findings by Hebeish & Sharaf (2015), who reported a similar absorbance peak at 960 cm^−1^ in their study on CMC-DADMAC copolymer hydrogels prepared *via* graft polymerisation.^40^

In the spectrum of CMC, intense bands at 1588.70 cm^−1^ and 1413.06 cm^−1^ are attributed to the asymmetric and symmetric stretching vibrations of the –COO^−^ group, originating from CH_2_COONa groups introduced during carboxymethylation with SMCA in the presence of NaOH.

Following both cationisation and carboxymethylation, crosslinking was carried out using ECH to synthesise the CC/CMC hydrogel. The FT-IR spectrum in Fig. 8c confirms successful crosslinking, as evidenced by the appearance of new and slightly shifted peaks. The band observed at 1644.09 cm^−1^, also contributed to the asymmetric stretching of carboxylate ions (COO^−^). The shift in the COO^−^ asymmetric band from 1588 cm^−1^ (pure CMC) to 1644 cm^−1^ (hydrogel) is attributed to new chemical interactions formed during crosslinking. This shift may be due to intermolecular interactions and hydrogen bonding effects after crosslinking with ECH.^41^ Similar results were reported by Yang *et al.* (2011), who observed a peak at 1608 cm^−1^ in CMC hydrogel beads after ECH crosslinking.^42^

Additionally, new peaks observed at 2913.07 cm^−1^ and 2879.77 cm^−1^ are attributed to the asymmetric and symmetric stretching vibrations of methylene groups (CH_2_), further confirming the integration of ECH into the hydrogel structure. These findings align with those of Ciolacu *et al.* (2022), who reported similar bands at 2930 cm^−1^ and 2873 cm^−1^ in microcrystalline cellulose hydrogels crosslinked with ECH.^43^ These bands were attributed to the same *ν*_asym_ (CH_2_) and *ν*_sym_ (CH_2_). Yan *et al.* (2009) also reported a peak at 2825 cm^−1^ related to CH_2_N vibrations in the synthesis of cationic hydroxypropyl cellulose hydrogels crosslinked with ECH for dye adsorption applications.^30^ Alongside FTIR analysis, FESEM results discussed in Section 3.6 offer additional confirmation of the effective crosslinking and successful formation of the CC/CMC hydrogel network.

### 3.4 XRD analysis of CC/CMC hydrogel

**Fig. 9:**
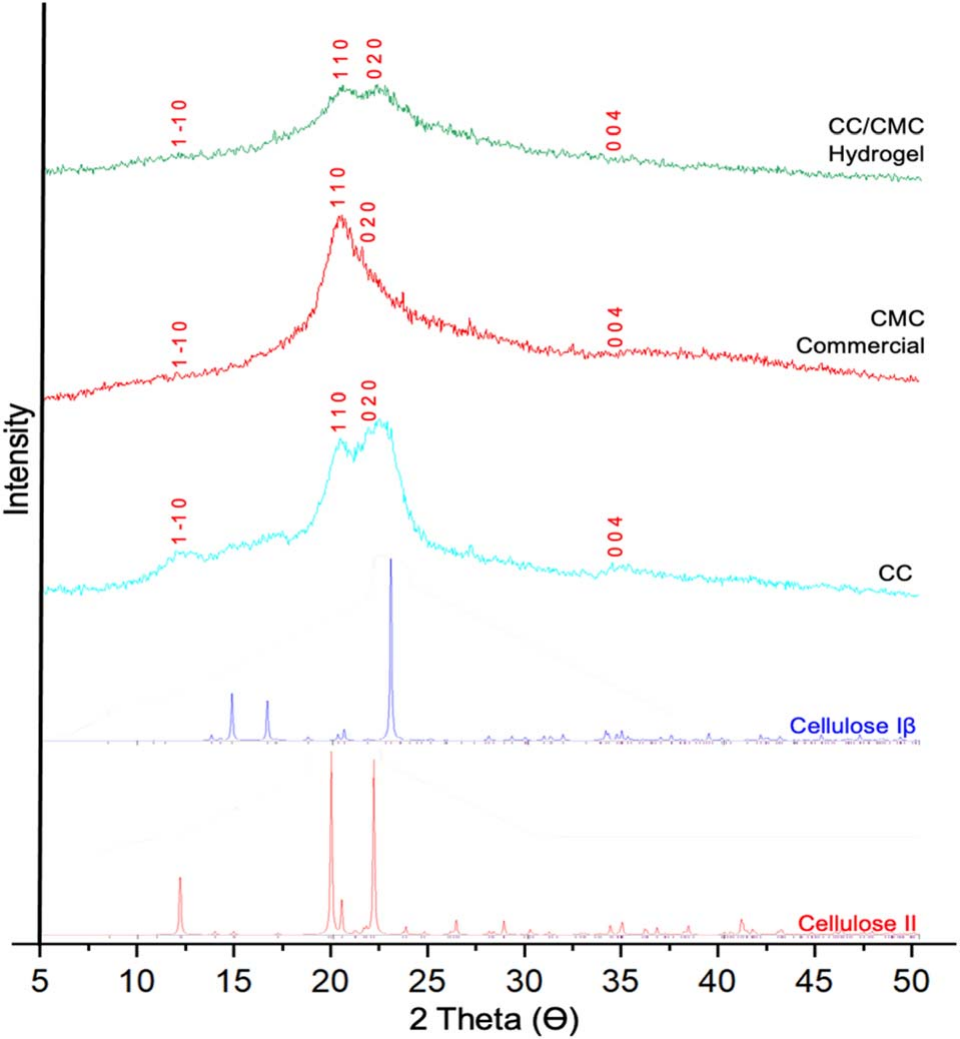
XRD diffractogram of CC, CMC and CC/CMC hydrogel with CC : CMC ratio of 4 : 6 with Miller indices provided by Mercury program of all calculated peaks.

The XRD diffractograms for CC, CMC and the CC/CMC hydrogel are presented in Fig. 9. For comparison, theoretical XRD patterns and corresponding Miller indices for cellulose Iβ and cellulose II, generated using the Mercury program, are also included. The interaction between cellulose crystallinity and its dissolution behaviour presents a notable challenge, primarily due to the coexistence of crystalline and amorphous regions within the cellulose matrix. The extensive hydrogen-bonding network in the crystalline domains significantly impedes dissolution in aqueous systems.

The XRD patterns revealed a complete transformation from cellulose I to cellulose II in all samples, as indicated by the absence of the characteristic cellulose Iβ diffraction peaks at 15° and 22.6°. Instead, all samples exhibited distinct peaks corresponding to the cellulose II polymorph at 11.93°, 20.32°, 22.08°, and 34.58°, which are associated with the (1–10), (110), (020), and (004) crystallographic planes, respectively. These reflections are in agreement with the standard cellulose II crystalline planes previously characterised by French (2014), who computationally modelled theoretical powder diffraction patterns for various cellulose allomorphs including Iα, Iβ, II, III_I_, and III_II_.^44^

The chemical modification processes led to the disruption of the strong inter- and intramolecular hydrogen bonds that define the cellulose crystalline structure. This disruption accounts for the observed reduction in crystallinity and contributes to enhanced water solubility, facilitating subsequent chemical transformations for various functional applications.

Mohkami & Talaeipour (2011), in their study on carboxylated and carboxymethylated fibres, similarly reported that treatment with NaOH disrupted the hydrogen bonding within cellulose crystalline regions, as evidenced by reduced crystallinity in XRD profiles.^45^

Among the analysed samples, CC/CMC hydrogel exhibited broader diffraction peaks, particularly around 20° and 22°, suggesting a higher degree of amorphous character. This increased amorphicity is attributed to the crosslinking process, which disrupts the molecular regularity and transforms the structure into a more disordered cellulose II form. The incorporation of a crosslinking agent introduces covalent bonds between CC and CMC chains, leading to the formation of a three-dimensional polymer network that enhances the hydrogel's functional properties.

To further understand the impact of crosslinking on the crystalline structure, Ramos Estevam *et al.* (2023) studied cellulose hydrogels prepared with epichlorohydrin (ECH) under various crosslinking and drying conditions.^46^ Their XRD results revealed peaks at approximately 13° and 22°, indicative of a semi-crystalline material with significant amorphous regions, consistent with the structural characteristics observed in the CC/CMC hydrogel in this study.

### 3.5 TG analysis of CC/CMC hydrogel

**Fig. 10:**
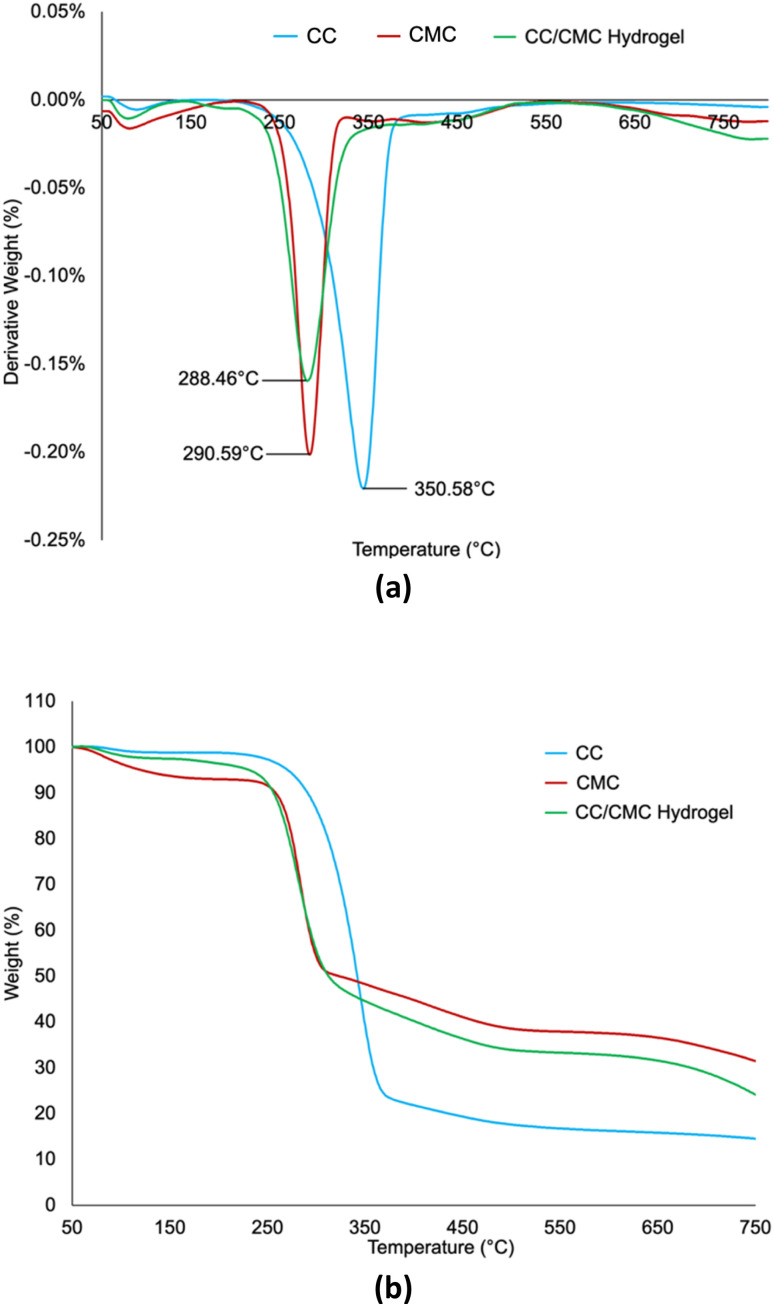
(a) TGA and (b) DTG thermograms of CC, CMC and CC/CMC hydrogel with CC : CMC ratio of 4 : 6.

The summary of thermal degradation analysis for CC/CMC hydrogel from TGA and DTG is tabulated in Table 6 while the thermograms are shown in Fig. 10(a) and Fig. 10(b), respectively. The thermograms show there are two decomposition stages for CC and three decomposition stages for CMC and CC/CMC hydrogel.

**Table 6:** Percentage of weight loss, decomposition temperature range and maximum rate decomposition temperature of CC, CMC and CC/CMC hydrogel^a^

Sample		Decomposition stage			Residue (%)
		1	2	3	
CC	Temperature (°C)	55.11–196.95	197.93–546.61	—	16.91
	Weight loss (%)	1.20	81.94	—	
	*T*_max_ (°C)	90.93	350.58	—	
CMC	Temperature (°C)	58.43–216.56	217.05–522.99	526.39–801.67	27.89
	Weight loss (%)	6.90	54.78	10.28	
	*T*_max_ (°C)	85.15	290.59	—	
CC/CMC hydrogel	Temperature (°C)	60.46–139.83	143.36–522.13	524.07–802.94	17.69
	Weight loss (%)	2.59	63.93	15.94	
	*T*_max_ (°C)	83.09	288.46	765.18	

a *T*_max_ – Maximum rate of decomposition temperature.

Carbonisation refers to thermal decomposition resulting in weight loss due to the degradation of various components such as moisture, carbohydrate polymers, and volatile substances in cellulose derivatives. The first decomposition stage represented by a sigmoidal shape in Fig. 10(a) corresponds to the release of bound water or surface moisture.

The second major decomposition phase, between 143 °C to 546 °C, marks the crucial degradation where significant transformations occur in the cellulose matrix. This stage, occurring above 200 °C, is characterised by weight loss ranging from 54% to 82%, due to dehydration, depolymerisation, decarboxylation, glycosidic bond cleavage and degradation of crystalline regions. These thermal events reflect the decomposition of the carbohydrate backbone, as supported by studies from Mohtar *et al.* (2017) and Tudorachi *et al.* (2012).^47,48^

The third decomposition stage, observed at temperatures from 524 °C to 802 °C, corresponds to the oxidation of partially degraded cellulose and charring. This final degradation phase reflects the carbonisation of residual material. Similar behaviour was noted by Lakshmi *et al.* (2017) and Zhang *et al.* (2015), who associated high-temperature weight loss with charring processes in seaweed- and linter-derived cellulose, respectively.^49,50^

Fig. 10(b) shows the DTG curves indicating the rate of weight loss and the corresponding temperature at maximum degradation (*T*_max_). In the first decomposition stage, all samples exhibited *T*_max_ values between 83-91 °C, indicating minor weight loss associated with moisture evaporation. Although the CC/CMC hydrogel sample was freeze-dried prior to analysis, a slight weight reduction of 2.59% was still observed, compared to 1.2% for CC and 6.9% for CMC. This small loss suggests the presence of tightly bound water molecules that remained within the polymeric network and were released during the initial heating stage. Bedane *et al.* (2015) categorised water interactions in cellulose as free, freezing-bound, and non-freezing-bound. Free water evaporates below 60 °C, while tightly bound water is released primarily around 90 °C, with some persisting up to 150 °C.^51^ Therefore, despite freeze-drying, the minor weight loss observed in the CC/CMC hydrogel corresponds to the removal of residual bound water molecules entrapped within the hydrogel matrix rather than from free moisture, supporting the use of a freeze-dried sample in this analysis. Odziomek *et al.* (2023) reported that freeze-dried hydrogels for medical applications exhibited a slight mass loss during the initial stage of TG decomposition, corresponding to the removal of some hydration.^52^

In second decomposition stage, CC showed the highest *T*_max_ at 350.58 °C with a weight loss of 81.94%, indicating superior thermal stability. This is due to its higher crystallinity, aligns with the findings of Oliveira *et al.* (2017), reported enhanced thermal resistance in cellulose fibres with elevated crystallinity.^53^ The structural rigidity conferred by the quaternary ammonium groups from DADMAC contributes to this thermal resilience. CMC exhibited a lower *T*_max_ of 290.59 °C, reflecting reduced thermal stability due to disruption of cellulose network. This trend was reported by El-Sakhawy *et al.* (2019), who observed thermal degradation in sugarcane bagasse-derived CMC following NaOH treatment, which induced amorphous structures.^54^ The *T*_max_ of the hydrogel was found to be 294.52 °C. This thermal stability may be attributed to its crystallinity, as confirmed by XRD data in Section 3.4, where a peak at 2*θ* = 22° was observed, indicating the presence of crystalline domains that require greater thermal energy to degrade. Poletto *et al.* (2014) also noted that such crystalline domains impede heat transfer, thereby enhancing thermal stability.^55^

Table 6 highlights that CC with *T*_max_ of 350.58 °C and residual mass of 16.91%, exhibits the highest thermal stability. The presence of quaternary ammonium groups forms a rigid polymeric structure, contributing to its resistance to thermal degradation. This finding is consistent with results from Yang *et al.* (2019), who reported increased residue content in CC monoliths modified with trimethylammonium groups.^56^ CMC and CC/CMC hydrogel displayed comparable *T*_max_ ranging from 288 °C to 290 °C. However, the residue analysis revealed slight differences in thermal stability. CMC yielded a higher residue than the CC/CMC hydrogel, suggesting marginally greater stability.

### 3.6 FESEM-EDX analysis of CC/CMC hydrogel

**Fig. 11:**
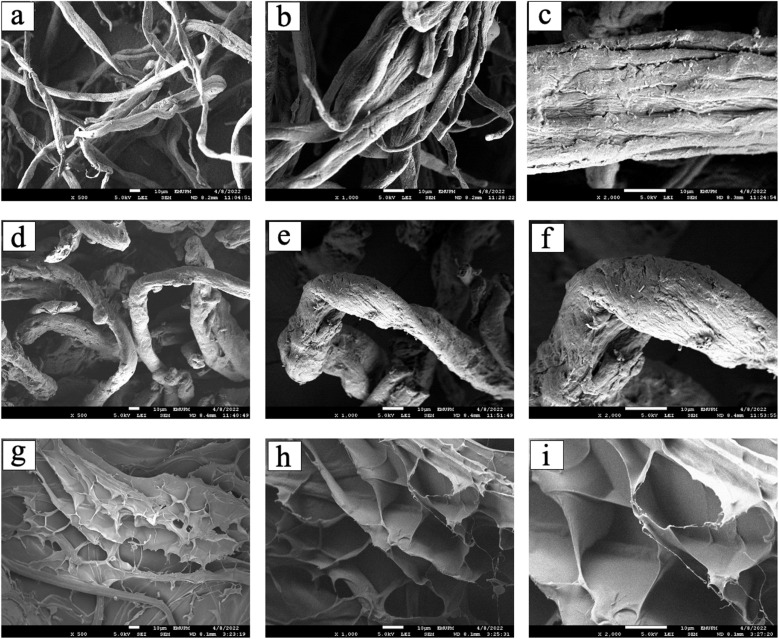
FESEM micrographs of (a–c) CC, (d–f) CMC and (g–i) CC/CMC hydrogel at magnifications of 500X, 1000X and 2000X.

**Fig. 12:**
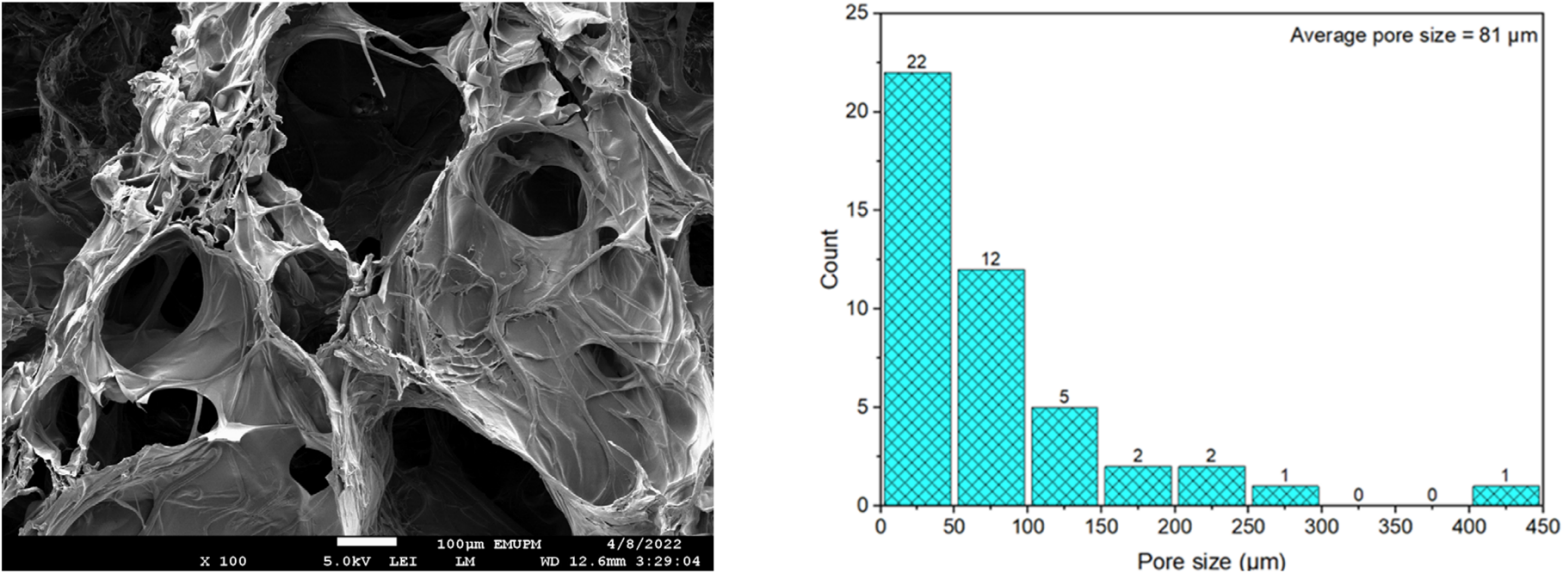
FESEM micrograph at X100 magnification and histogram of pore size distribution for CC/CMC hydrogel.

**Fig. 13:**
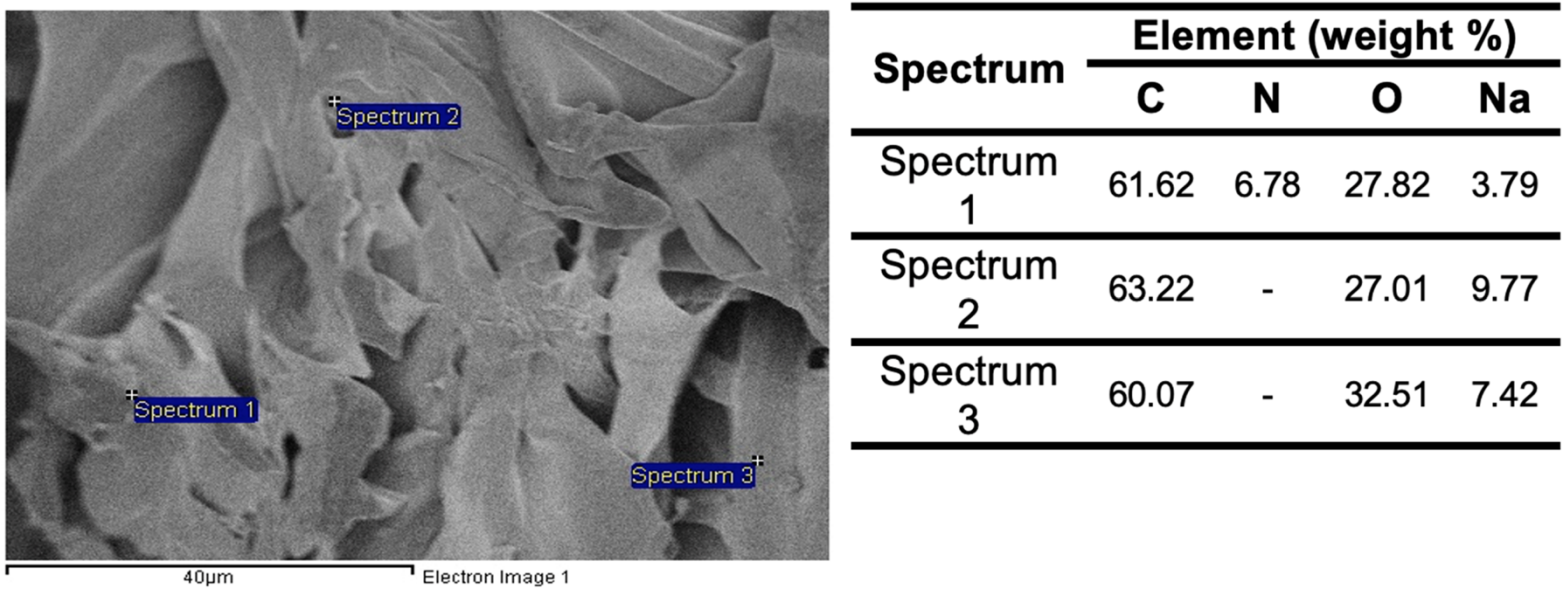
FESEM-EDX micrograph of CC/CMC hydrogel with percentage of elemental analysis.

The FESEM combined with EDX analysis was employed to investigate the morphology and surface elemental composition of all cellulose derivative samples. Fig. 11 presents the micrographs of CC, CMC and CC/CMC hydrogel at magnifications of 500X, 1000X and 2000X. Meanwhile, Fig. 12 shows the FESEM image at 100X magnification and the pore size distribution of the hydrogel. Fig. 13 illustrates the elemental composition obtained *via* EDX for CC and CC/CMC hydrogel.

In Fig. 11(a–c), high-resolution FESEM imaging reveals surface morphological features of CC after modification *via* cationisation using DADMAC in the presence of NaOH. The resulting CC sample exhibits a wrinkled, woven texture with visible surface cavities, which attributed to the removal of inorganic inclusion such as silica bodies and structural arrangement due to the transformation from cellulose I to cellulose II. Silica bodies, also known as opaline phytoliths, are typically located in the epidermis or sheath cells of vascular bundles, where they provide structural, protective, and physiological functions in plant tissues. Their diameters usually range from 6 to 10 µm, with an average of about 7.8 µm; however, in the present findings, they ranged from 15 to 23 µm. This variation arises from differences in morphology, size, location, and composition among plant species and genera.^57^ This leads to a roughened fibre surface. Similar features have been reported by Noori *et al.* (2021), while Aguado *et al.* (2018) observed damaged surfaces in CC fibres derived from crop residues following the cationisation reactions.^58,59^

The CMC in Fig. 11 (d–f) displays a rough surface morphology from the carboxymethylation reaction in the presence of NaOH under Williamson ether synthesis conditions. NaOH serves both as a swelling agent and as an alkaline medium that facilitates the diffusion of the etherifying agent into the cellulose structure. The consequent structural disruption produces a wrinkled and uneven surface. This observation is consistent with the findings of Lawal *et al.* (2007), who noted surface alterations in carboxymethylated starches exposed to strong alkaline conditions.^60^

Fig. 11 (g–i) present the cross-sectional morphology of the CC/CMC hydrogel, revealing a highly porous and interconnected macroporous structure. The observed porosity is attributed to the crosslinking reaction between the hydroxyl groups of cellulose derivatives and the epoxy groups of ECH, forming a stable three-dimensional network. Micrographs of hydrogels formulated with a 4 : 6 ratio of CC to CMC demonstrate varied porosity even in the unswollen state.

The formation of pores is influenced by phase separation during freeze-drying, where ice crystals develop within the hydrogel matrix and are subsequently sublimated, leaving voids. Moosavi *et al.* (2020) reported a similar mechanism. These pores enhance the ability of the hydrogel to absorb fluids by providing greater surface area and internal space.^61^ Analysis of FESEM micrograph and corresponding histogram in Fig. 12 confirms the average pore size of the CC/CMC hydrogel to be approximately 81 µm, with pore size extending up to 450 µm. The range of the pore sizes, delineated by sheet-like and ultra-thin walls, aligns with the structures described by Alam *et al.* (2019) in their study of superabsorbent cellulose hydrogels prepared from bleached softwood kraft pulp.^62^ Comparable open-porous morphologies with pore diameters in the range of 300–600 µm were reported in their study, whereas the CC/CMC hydrogel in the present work exhibited a finer average pore size of 81 µm and correspondingly attained a high swelling capacity of 116.94 g g^−1^. This enhanced water update is attributed to the increased internal volume created by its porous architecture.

Elemental composition analysis of the hydrogel sample *via* EDX is shown in Fig. 13. The analysis revealed a nitrogen content of 4.95% at Spectrum 2, indicating successful incorporation of nitrogen-bearing functional groups, likely introduced during the cationisation modification. This supports the effective chemical functionalisation and crosslinking of the hydrogel network.

## 4. Conclusions

In this study, CC/CMC hydrogel was successfully optimised from the CC synthesised from OPEFB and incorporated with CMC to form a crosslinked hydrogel using ECH. The application of RSM based on CCD model enables the identification of optimal formulation conditions, particularly the CC : CMC ratio of 4 : 6, ECH concentration of 6.06% and a reaction temperature of 60 °C. Under these conditions, the CC/CMC hydrogel exhibited an optimal gel content of 20.95% and an impressive degree of swelling of 116.94 g g^−1^. Structural analysis confirmed chemical modification and successful network formation, with FT-IR, XRD and TGA results highlighting the introduction of functional groups, transformation to amorphous cellulose II and enhanced thermal stability of the material. FESEM-EDX results supported these findings by revealing a macroporous structure and elemental incorporation of nitrogen from the cationic groups. Overall, this study demonstrates a green and efficient pathway for converting agricultural biomass waste into value-added, biodegradable hydrogels with promising potential. These hydrogels can be tailored for diverse industrial applications, including controlled-release systems, water retention, and environmental remediation.The integration of statistical modelling offers a robust and reproducible platform for hydrogel optimisation in future materials design.

## Author contributions

Conceptualization, N. Z.; methodology and design, N. Z., and N. F. A.-Z. T. M.; software, N. F. A.-Z. T. M.; formal analysis and investigation, N. Z., N. F. A.-Z. T. M., S. K., and H. A.; writing—original draft preparation, N. F. A.-Z. T. M.; writing—review and editing, N. Z. and N. F.A.-Z. T. M.; supervision, N. Z., S. K., and H. A. All authors have read and agreed to the published version of the manuscript.

## Conflicts of interest

There are no conflicts to declare.

## Data Availability

The authors confirms that the data supporting the findings of this study are available within the article.
